# Genome-wide selection signatures reveal widespread synergistic effects of two different stressors in *Drosophila melanogaster*

**DOI:** 10.1098/rspb.2022.1857

**Published:** 2022-10-26

**Authors:** Claire Burny, Viola Nolte, Marlies Dolezal, Christian Schlötterer

**Affiliations:** ^1^ Institut für Populationsgenetik, Vetmeduni Vienna, Veterinärplatz 1, Vienna 1210, Austria; ^2^ Vienna Graduate School of Population Genetics, Vetmeduni Vienna, Vienna 1210, Austria; ^3^ Plattform Bioinformatik und Biostatistik, Vetmeduni Vienna, Vienna 1210, Austria

**Keywords:** experimental evolution, *Drosophila melanogaster*, parallel evolution, local adaptation, temperature adaptation

## Abstract

Experimental evolution combined with whole-genome sequencing (evolve and resequence (E&R)) is a powerful approach to study the adaptive architecture of selected traits. Nevertheless, so far the focus has been on the selective response triggered by a single stressor. Building on the highly parallel selection response of founder populations with reduced variation, we evaluated how the presence of a second stressor affects the genomic selection response. After 20 generations of adaptation to laboratory conditions at either 18°C or 29°C, strong genome-wide selection signatures were observed. Only 38% of the selection signatures can be attributed to laboratory adaptation (no difference between temperature regimes). The remaining selection responses are either caused by temperature-specific effects, or reflect the joint effects of temperature and laboratory adaptation (same direction, but the magnitude differs between temperatures). The allele frequency changes resulting from the combined effects of temperature and laboratory adaptation were more extreme in the hot environment for 83% of the affected genomic regions—indicating widespread synergistic effects of the two stressors. We conclude that E&R with reduced genetic variation is a powerful approach to study genome-wide fitness consequences driven by the combined effects of multiple environmental factors.

## 1. Introduction

Ecological genetics aims to characterize the interaction of organisms with their environment. Of particular interest is the characterization of adaptive responses, which are specific to a given habitat. Many approaches have been pursued to study the genetic basis of local adaptation [1–5]. Allele frequency differences between populations from different environments are particularly powerful to test for correlation between genetic variation and environmental variables [6,7] and are widely applied to the analysis of clinal variation [8–12]. Despite being conceptually appealing, this approach faces several challenges. Historical demographic events, such as bottlenecks or admixture, may generate confounding signals, possibly resulting in false positives/negatives [13–17]. Furthermore, estimating covariance between allele frequencies and environment is difficult as (i) identifying and/or measuring the relevant environmental variables is challenging since many abiotic factors are correlated [18,19] and (ii) selection can vary over time (e.g. [20–22]).

Experimental evolution, in particular laboratory natural selection, allows the study of adaptive responses in a controlled laboratory environment [23–26]. Exposing a mixture of genotypes to a monitored stressor, the adaptive response can be measured through time in replicate populations, combined with next-generation sequencing (evolve and resequence (E&R); [27–29]). While many experimental evolution studies rely on truncating selection to determine the genotypes contributing to the next generation [27,30–33]), laboratory natural selection builds on fitness differences between genotypes upon exposure [24] and hence provides a closer fit to adaptation and competition processes occurring in the wild [34].

E&R studies typically focus on the genomic response to a single stressor, such as temperature [35–37], food quality [38] and developmental time [39]. Nevertheless, it is well-documented that exposure to multiple stressors frequently uncovers non-additive effects [40–43]. This has important implications for E&R studies which start from a large number of freshly collected flies [31,44–47] to reduce the impact of a moderate number of recombination events during the experimental evolution phase [48]. Natural populations are not adapted to laboratory conditions, which implies that the evolutionary response of such E&R studies includes adaptation to laboratory conditions and to the specific treatment of the experiment. Experimental evolution studies contrasting populations originating from the same founder population, but evolved at different treatments make the implicit assumption that laboratory adaptation can be ignored because the evolved flies share the same laboratory conditions. This assumption holds only when laboratory adaptation does not influence the experimental treatment.

Here, we study the influence of multiple stressors on the adaptive genomic response by testing the adaptive response to stress imposed by laboratory conditions at two different temperature regimes. We used 18°C, a putatively non-stressful temperature regime since the two founder genotypes of our experiment showed very similar gene expression profiles at 18°C [49,50]. By contrast, 29°C is a very stressful temperature regime, close to the maximal temperature at which *Drosophila melanogaster* populations can be maintained [51]. We avoided the complex selection responses which are typical of E&R experiments using many founder haplotypes by restricting the experiment to two founder genotypes. We recently showed that this design results in very pronounced, highly parallel selection responses across multiple replicates

Experimental evolution in the laboratory will always experience the selection pressure from culturing conditions, which precludes a fully nested design. Nevertheless, it is possible to distinguish selection responses that are driven by (i) laboratory adaptation only (same response in both temperature regimes), (ii) temperature only (response either only at one temperature or in the opposite direction), and (iii) temperature-specific laboratory adaptation (response in the same direction, but with different magnitude). At both temperature regimes we observed a very strong selection response across the entire genome. About one-third of the genomic regions responded either to temperature only, to laboratory conditions only, or exhibited a significant joint effect of both stressors. Our results demonstrate the importance of the combined effects of different environmental factors.

## 2. Material and methods

### (a) Experimental set-up

We used the Oregon-R and Samarkand strains inbred by Chen *et al*. [49], and maintained since then at room temperature. The three replicates of both experimental evolution cages were set up in parallel, each with a census size of 1500 flies and with an accidental starting frequency of 0.3 for the Oregon-R genotype (0.7 for the Samarkand genotype)—rather than 0.5, as described in [52]. Briefly, all replicates were then maintained for 20 generations at either constant 29°C or constant 18°C in dark conditions before sequencing. Three hundred adults were transferred every generation to one of five bottles for two days of egg laying. After egg laying, all adults were removed and frozen. The egg lay resulted in a high density of larvae. Hence, we transferred a mixture of larvae and food to two fresh food bottles. Adults collected 8–32 h after eclosure of the first flies from all bottles were mixed to avoid population substructure. A total of 300 adults from each vial started the next generation. While we started the two temperature regimes at the same time, owing to the slower development at 18°C, populations from this temperature regime reached generation 20 at a later time point.

### (b) DNA extraction, library preparation, sequencing

Whole-genome sequence data for the parental Oregon-R and Samarkand strains are available in [49]. The evolved replicates in generation F_20_ were sequenced using Pool-Seq: genomic DNA was prepared after pooling and homogenizing all available individuals of a given replicate in extraction buffer, followed by a standard high-salt extraction protocol [53]. For the samples in the 29°C experiment, barcoded libraries with a targeted insert size of 480 bp were prepared using the NEBNext Ultra II DNA Library Prep Kit (E7645L, New England Biolabs, Ipswich, MA) and sequenced on a HiSeq 2500 using a 2 × 125 bp paired-end protocol. The sequencing data from 29°C were taken from [52]. For the samples in the 18°C experiment, we used the same library preparation protocol, but with a target insert size of 280 bp, and 2 × 150 bp reads were sequenced on the HiSeq X Ten platform. Overlapping reads from read-pairs were clipped with bamUtils clopOverlap. Although differences in insert size can affect allele frequency estimates of single, single nucleotide polymorphisms (SNPs) [54], this does not affect our analysis because allele frequencies are estimated for many SNPs together.

### (c) Allele frequency tracking

We previously established a catalogue of parental SNPs [52]. Briefly, a parental SNP was defined as a (nearly) fixed difference between parental lines with a 0/0 (1/1) genotype in the Samarkand parent and 1/1 (0/0) genotype in the Oregon-R parent at the marker position, conditioning for a frequency of the alternate allele lower than 0.05 (if 0/0) or higher than 0.95 (if 1/1) for a final list of 465 070 SNPs; 401 252 and 63 818 SNPs on the autosomes and the X chromosome, respectively, equivalent to 1 SNP every 271 bp on the autosomes and 363 bp on X. The same processing and mapping steps were applied at 29°C and 18°C described in [52]. The allele frequency have been obtained after converting processed BAM files from pileup (*samtools mpileup -BQ0 -d10000*; v. 1.10; [55]) to sync files (using PoPoolation2 *mpileup2sync.jar*; [56]). We then tracked the allele frequency at F_20_ of the Oregon-R allele in three replicates at 29°C (replicates 1,2,3 in [52]) and three replicates at 18°C. The subsequent analyses have been performed with R (v. 4.0.4; [57]) and most panels have been generated with the ggplot2 R package [58]. We retained SNPs measured at both temperatures, leading to a total of 100,283, 89,929, 107,119, 103,760, 72, 63,766 SNPs on 2L, 2R, 3L, 3R, 4 and X. Because the average coverage at the marker SNPs differs between both temperatures (12, 11, 9× at 18°C and 123, 107, 133× at 29°C), we down-sampled the 29°C coverage values to 12× by drawing the coverage at each locus from a Poisson distribution of mean 12 and then applying binomial sampling with a sample size set to the sampled coverage to mimic Pool-Seq sampling noise [59]. In order to both limit noise in allele frequency measurements and to take linkage into account, the allele frequency values are averaged in non-overlapping windows of size *w* = 50, 250 or 500 SNPs for a total of 8021, 1603, 801 measurements on the autosomes (2 and 3) and 1275, 255, 127 on X for each window size respectively, where the last window of each chromosome, containing fewer than *w* SNPs. Windows of size *w* = 50, 250 or 500 SNPs correspond to 13.6 [12.8; 14.4], 67.8 [59.7; 76.0] and 135.6 [115.8; 155.4] kb on average for the autosomes and 18.2 [16.6; 19.7], 90.5 [81.4; 99.5] and 180.9 [162.1; 199.8] kb for X. The 95% confidence intervals have been obtained by the mean ± 1.96 standard error (s.e.). The main results are represented at 250-SNP level. A window position *i* is defined by its centre ((right-left)/2). By convention, if the Oregon-R allele frequency at F_20_ is higher (lower) than its initial frequency of 30% (70%), the Oregon-R (Samarkand) allele increased in frequency and the allele frequency change (AFC) is positive (negative).

### (d) Comparing the response between the 18°C and 29°C selection regimes

We classified the AFC of each window after 20 generations as non-significantly deviating from neutrality or presenting a selection signal. In order to test deviation from neutrality, we performed 100 neutral simulation runs using MimicrEE2 [60]. The neutral simulations mimic the experimental set-up, i.e. starting with 30% of Oregon-R flies over 1500 flies, using three replicates and the same marker SNPs providing the *D. melanogaster* recombination map [61] updated to version 6 of the reference genome using the Flybase online converter (https://flybase.org/convert/coordinates; accessed in July 2020). For each simulation run, we computed the average AFC over the three replicates per window. Per temperature and per chromosome, an empirical *p*-value per window *w* ($p_{w}^{18^{\circ }\mathrm{Cneutral}}$ or $p_{w}^{29^{\circ }\mathrm{Cneutral}}$) is calculated as the fraction of AFC values higher (lower) than the empirical AFC when the observed AFC is positive (negative) divided by the total number of average AFC values. We finally applied a Benjamini-Hochberg correction per chromosome ($p.{\mathrm{adj}}_{w}^{18^{\circ }\mathrm{Cneutral}}$ and $p{.\mathrm{adj}}_{w}^{29^{\circ }\mathrm{Cneutral}}$). If a window presents a selection signal, it either favours the same parental allele at both temperatures (with a change in magnitude or not) or different alleles—for example the Oregon-R allele at 29°C (${\mathrm{AFC}}_{w}^{29^{\circ }C}>0$) and the Samarkand allele at 18°C (${\mathrm{AFC}}_{w}^{18^{\circ }C}<0$). To check which scenario is more likely, we fitted a simple linear model (LM) for each window w, with AFC as response and temperature as fixed categorical explanatory factor, where $\alpha_{w}^{\mathrm{intercept}}$ corresponds to 18°C-reference level and $\alpha_{w}^{\mathrm{temperature}}$ is the contrast between 29°C and 18°C. We extracted the corresponding *p*-value ($p_{w}^{\mathrm{LM}}$) and applied a Benjamini-Hochberg correction per chromosome on the non-neutral windows ($p.{\mathrm{adj}}_{w}^{\mathrm{LM}}$). A significant window is classified as displaying a change in magnitude with the temperature favouring the same parental allele ($\alpha_{w}^{\mathrm{intercept}}$ and $\alpha_{w}^{\mathrm{temperature}}$ of same sign) or a different allele ($\alpha_{w}^{\mathrm{intercept}}$ and $\alpha_{w}^{\mathrm{temperature}}$ of different sign). For a given false discovery rate (FDR) threshold, a genomic window w is then classified in one of the following six classes: ‘drift only’, ‘change 18°C only’, ‘change 29°C only’, ‘no temperature effect’, ‘different magnitude’ and ‘different direction’ (see the electronic supplementary material, table S1 for logical conditions on windows affectation to each class). We then recorded the fraction of windows affected in each of the six classes for different values of FDR (5%, 10%, 15%) per chromosome and averaged genome-wide. We also computed the autocorrelation per chromosome and per replicate using the *acf* R function; the autocorrelation at a given step *k* is defined as the correlation between windows at positions *i* and *i + k*, where *k* is called the lag. We eventually recorded the distance where a significant decrease in autocorrelation at a 5% threshold (below 1.96/√*n*, *n* the number of windows), i.e. a rough proxy of linkage equilibrium, is reached.

## 3. Results

We exposed two genotypes, Samarkand and Oregon-R, to two different environmental stressors, laboratory adaptation and temperature. Two E&R experiments shared the same laboratory environment but differed in temperature regime. Three replicate populations were maintained for 20 generations at either 18°C or 29°C. Genome-wide allele frequencies of genotype-specific marker SNPs were determined by Pool-Seq [28]. Because genotype-specific alleles start at the same frequency in all replicates and only few recombination events were expected during the experiment, we averaged the allele frequencies in non-overlapping windows of 250 consecutive SNPs to obtain reliable allele frequency estimates. This strategy is supported by the high autocorrelation of neighbouring SNPs, up to a distance of 6.7 Mb (electronic supplementary material, figure S1). We inferred selection by contrasting the allele frequencies of the Oregon-R genotype at the start of the experiment (30%) to those after 20 generations, relative to simulated frequency changes under neutrality. A positive AFC indicates that the Oregon-R allele increased in frequency.

**Figure 1.**
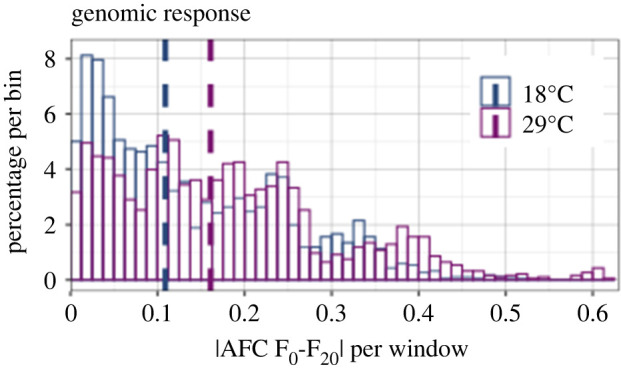
Differences between the parental genotypes at 18°C (blue) and 29°C (purple). Histograms of absolute allele frequency change of the Oregon-R allele between F_0_ and F_20_ (|AFC F_0_-F_20_|) for non-overlapping windows of 250 SNPs.

**Figure 2.**
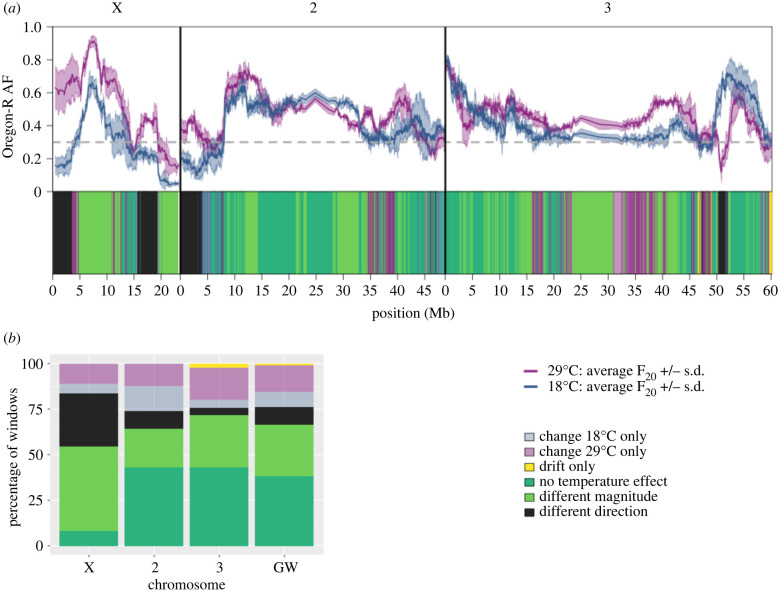
(*a*) Top: genome-wide allele frequencies (AF) after evolving for 20 generations at two temperature regimes. The frequency of the Oregon-R allele is averaged for non-overlapping windows of 250 SNPs (solid line) ± one standard deviation (shaded area) at 18°C (blue) and 29°C (purple). Bottom: each window is classified (see Methods) in one of the six colour-coded classes depending on the AFC between 18°C and 29°C: change at 18°C only (light blue), change at 29°C only (light purple), drift only (yellow), no temperature effect (dark green), different AFC magnitude but same direction of effect (light green), opposite alleles increase at 18°C and 29°C (black). (*b*) Percentage of the genomic windows per class (defined for an FDR threshold of 10% per chromosome and averaged genome-wide (GW)).

After 20 generations marked allele frequency changes occurred at both temperature regimes (figure 1). The three replicate populations of each temperature regime showed a strikingly parallel selection response as indicated by the shaded area corresponding to +/− one standard deviation around the mean of the three replicates (figure 2a). Overall, Oregon-R alleles were more likely to increase in frequency than Samarkand alleles, with 90% and 80% of the windows displaying positive AFC at 18°C and 29°C respectively.

Given that the *Drosophila* populations were adapting to two different environmental stressors, laboratory environment and temperature, it is possible to evaluate their individual and joint effect on the selection response across the entire genome. We characterized the selection response by classifying windows changing more in frequency than expected under neutrality in each of the temperature regimes (figure 2a,*b*, electronic supplementary material, table SI 1). On the one hand, the direction of the selection response, i.e. the increase in frequency of the Oregon-R or Samarkand alleles, differed for 10% of the windows between the two temperatures (figure 2, black). Eight per cent (figure 2, light blue) and 14% (figure 2, purple) of the windows displayed a significant allele frequency change relative to drift, only at either 18°C or 29°C respectively. On the other hand, a similar allele frequency change was observed for 38% (figure 2, dark green) of the windows in the two experiments, which we attribute to laboratory adaptation only. In total, roughly two-thirds of the genome responded only to one of the two environmental stressors. Nevertheless, a remarkablylarge fraction of windows showed a significant combined effect of the two environmental stressors. Twenty-eight per cent (figure 2, light green) changed in the same direction, but at a different magnitude between temperatures. This pattern of frequency change indicates that temperature modulates the adaptive response to the selective force common to both experiments. For most of these windows (83%), the most extreme allele frequency change was observed at 29°C, which may suggest that the two stressors, temperature and laboratory environment, act synergistically. Only a small fraction (1%, figure 2, yellow) of windows did not change in frequency beyond what is expected by drift in either treatment. Qualitatively similar results were obtained when the comparison between the two temperature regimes was performed for single SNPs or averaged across windows of 50, 250 and 500 SNPs as well as with different FDR thresholds (electronic supplementary material, figure S2).

## 4. Discussion

We studied the selective impact of two different environments on a genomic scale by combining laboratory and temperature adaptation. Contrary to the recommended design for E&R studies [48], which facilitate the identification of a moderate number of selection targets occurring at sufficiently high starting frequencies, we did not use a large number of founder genotypes. Rather, we restricted the variation to only two different founder genotypes, as in experimental evolution with yeast (e.g. [62]). The advantage of this experimental design is that all selection targets have the same starting frequency and a more parallel selection response is expected because polygenic traits have fewer selection targets contributing to a new trait optimum [63,64].

We found pronounced selection responses, which fall into three classes: (i) temperature-specific (allele frequency change either only at one temperature or in the opposite direction), (ii) laboratory adaptation (parallel selection with similar intensities in the two temperature regimes), and (iii) joint contribution of both environmental factors—28% of the genomic windows responded in the same direction, but to a different extent.

Temperature-specific adaptation implies that temperature uncovers fitness differences between genotypes. A total of 14% of the genomic windows responded only at 29°C and 8% were private to 18°C, a pattern consistent with conditional neutrality [65]. The selection responses private to 18°C indicate that even at an assumed benign temperature [49], selection occurs—highlighting the challenge of performing control experiments for temperature adaptation. In 10% of the windows, different alleles were favoured at each temperature regime. The observation that roughly one-third of the temperature-specific responses are selected in the opposite direction differs from an E&R study in *Drosophila simulans*, which found no shared haplotype block that was selected in the opposite direction in hot and cold environments [66]. Given the broad genomic regions affected in our study, it is not possible to decide whether the same allele experiences selection in the opposite direction or different alleles on the two genotypes show conditional neutrality (i.e. respond to one temperature regime only). Additional generations as well as a larger population size could facilitate the uncoupling of causative variants and passenger alleles to improve resolution [67,68].

Laboratory adaptation is an umbrella term for stressors that can be attributed to the experimental laboratory set-up [69–71]. Examples of such factors are adaptation to high larval density/early fertility [72,73], sexual selection [74] and adaptation to the laboratory food [40,75,76]. With about one-third of the genomic windows showing a parallel selection response at both temperature regimes, laboratory adaptation was an important factor in this study. This observation is highly consistent with a series of publications, which focused on laboratory adaptation in *Drosophila subobscura* and found strong phenotypic and genomic responses [70,77]. The abundant evidence for laboratory adaptation in this study contrasts the observation of an E&R study in *D. simulans*, which found no shared haplotype block moving in the same direction between hot and cold temperatures [66].

Of particular interest is the significant difference in allele frequency change for 28% of the windows with parallel selection signatures, because it suggests that laboratory adaptation is driving the parallel response and temperature modulates the strength of selection. Adaptation to larval density may be an excellent candidate driving this laboratory adaptation because we maintained the populations at high, but not well-controlled, larval densities. Higher larval density does not only increase competition [78] but also interactions between larval density and heat stress survival [79] as well as body size [80] and locomotor activity [81] were previously detected. Particularly interesting was the observation that in 83% of the windows the more stressful temperature environment resulted in a larger allele frequency change, which indicates that synergistic effects of the two stressors, high temperature and laboratory adaptation, are widespread and involve multiple genomic locations.

Because laboratory experiments cannot fully match natural conditions, it is not possible to conduct these experiments in a full factorial design—we can only modulate the temperature under laboratory conditions, but not in the natural environment. This implies that our design cannot distinguish between additive and interaction effects of temperature *per se* and laboratory adaptation. It is conceivable that the observed selection responses are driven by temperature only. This is undoubtedly the case for temperature-specific responses, where the populations responded in the opposite direction in the two temperature regimes. It is, however, less obvious that temperature adaptation alone could drive the same selection response in the two temperature regimes. Hence, we consider it more likely that temperature and laboratory adaptation operated jointly in our experiments. It is also possible that laboratory adaptation may differ at the two temperature regimes. For example, the microbiome composition may change with temperature [82,83] or properties of the food may also vary. Nevertheless, well-known components of the laboratory culture conditions are shared between the two temperatures, such as high larval density and implicit selection for early fecundity, which make a temperature-specific laboratory adaptation unlikely.

Selection responses driven by multiple selection factors can be problematic for the interpretation of the selection signatures. Experiments contrasting ancestral and evolved populations cannot distinguish between laboratory adaptation and selection driven by the focal factor (temperature in our study). When populations are compared, which evolved towards two different focal environments (here, different temperatures), the influence of laboratory adaptation is less severe: selection responses with the same direction and magnitude will not be seen in this contrast. Parallel selection responses that differ in magnitude will be interpreted as a pure temperature effect. An experimental design, which does not only include populations evolved in two different focal conditions (i.e. different temperatures), but also the ancestral founder populations can distinguish between laboratory adaptation, adaptation to focal factor and combined effects. Nevertheless, if laboratory adaptation interacts with temperature (or other focal factors), it is possible that small differences in laboratory environment (e.g. food recipe) may result in a different selection response. This may contribute to difficulties in replicating temperature-associated effects.

Genotype-specific deleterious mutations are an alternative explanation for the shared directional selection response at 18°C and 29°C. Since the two parental strains were maintained at small effective population sizes for many generations, it is conceivable that the influence of deleterious alleles is more pronounced than for genotypes freshly collected from the wild. The selection signatures may thus also reflect fitness costs of deleterious combinations of parental alleles that can be detected when the two competing genotypes are maintained at a large population size. The observation that temperature stress can both increase and decrease the selection response is consistent with previous studies on deleterious mutations [84]. While the selection response was frequently found to be positively correlated with stress level (e.g. [85,86]), also the opposite pattern has been observed [87,88]. Since we cannot determine how much of the parallel selection response can be attributed to deleterious mutations, it is important to realize that we probably overestimate the influence of laboratory adaptation.

One important limitation of this study is the pronounced linkage disequilibrium in the founder population. During 20 generations, too few recombination events occur to break the association between neighbouring windows. This is indicated by autocorrelation of allele frequency up to 8 Mb. Thus, even though our analyses are based on windows of 250 neighbouring SNPs, neighbouring windows are still highly correlated. This implies that neighbouring windows may exhibit a selection response owing to linkage, rather than owing to an independent selection target. Different selection intensities will also determine the size of the genomic region affected, leading to a complex interplay between linkage, direction of selection and selection strength. Therefore, the number of windows showing a given selection response may not be an accurate reflection of the number of selection targets with a given behaviour. Nevertheless, the prevailing effects of temperature and laboratory adaptation on fitness should be robust against the effects of linkage.

We conclude that E&R experiments starting with strongly reduced genetic variation can provide a powerful approach to study adaptation, in particular when experiments are performed on an environmental gradient (i.e. multiple different temperatures). This set-up provides new insights into adaptation, in particular when the E&R experiments are performed for more than only 20 generations, since additional generations provide more opportunity for recombination and the selection targets can be characterized with a higher resolution.

## Data Availability

The sequencing data underlying this article are available in the European Nucleotide Archive (ENA) at https://www.ebi.ac.uk/ena/browser/view/, and can be accessed with PRJEB46805 from [52] (29°C) and PRJEB55541 for new data (18°C) specifically generated for this study. All scripts (command lines and data analysis) and final files underlying this article are available in Zenodo at https://dx.doi.org/10.5281/zenodo.7015011 [89]. Additional table and figures underlying this article are available in the online electronic supplementary material [90].
